# Comparative Analysis of Inflammatory Cytokine Release and Alveolar Epithelial Barrier Invasion in a Transwell^®^ Bilayer Model of Mucormycosis

**DOI:** 10.3389/fmicb.2018.03204

**Published:** 2019-01-08

**Authors:** Stanislav Belic, Lukas Page, Maria Lazariotou, Ana Maria Waaga-Gasser, Mariola Dragan, Jan Springer, Juergen Loeffler, Charles Oliver Morton, Hermann Einsele, Andrew J. Ullmann, Sebastian Wurster

**Affiliations:** ^1^Division of Infectious Diseases, Department of Internal Medicine II, University Hospital of Würzburg, Würzburg, Germany; ^2^Department of Surgery I, University Hospital of Würzburg, Würzburg, Germany; ^3^School of Science and Health, Western Sydney University, Sydney, NSW, Australia; ^4^Department of Infectious Diseases, University of Texas MD Anderson Cancer Center, Houston, TX, United States

**Keywords:** mucormycosis, alveolar epithelium, *in vitro* model, cytokines, dendritic cells

## Abstract

Understanding the mechanisms of early invasion and epithelial defense in opportunistic mold infections is crucial for the evaluation of diagnostic biomarkers and novel treatment strategies. Recent studies revealed unique characteristics of the immunopathology of mucormycoses. We therefore adapted an alveolar Transwell^®^ A549/HPAEC bilayer model for the assessment of epithelial barrier integrity and cytokine response to *Rhizopus arrhizus, Rhizomucor pusillus*, and *Cunninghamella bertholletiae*. Hyphal penetration of the alveolar barrier was validated by 18S ribosomal DNA detection in the endothelial compartment. Addition of dendritic cells (moDCs) to the alveolar compartment led to reduced fungal invasion and strongly enhanced pro-inflammatory cytokine response, whereas epithelial CCL2 and CCL5 release was reduced. Despite their phenotypic heterogeneity, the studied Mucorales species elicited the release of similar cytokine patterns by epithelial and dendritic cells. There were significantly elevated lactate dehydrogenase concentrations in the alveolar compartment and epithelial barrier permeability for dextran blue of different molecular weights in Mucorales-infected samples compared to *Aspergillus fumigatus* infection. Addition of monocyte-derived dendritic cells further aggravated LDH release and epithelial barrier permeability, highlighting the influence of the inflammatory response in mucormycosis-associated tissue damage. An important focus of this study was the evaluation of the reproducibility of readout parameters in independent experimental runs. Our results revealed consistently low coefficients of variation for cytokine concentrations and transcriptional levels of cytokine genes and cell integrity markers. As additional means of model validation, we confirmed that our bilayer model captures key principles of Mucorales biology such as accelerated growth in a hyperglycemic or ketoacidotic environment or reduced epithelial barrier invasion upon epithelial growth factor receptor blockade by gefitinib. Our findings indicate that the Transwell^®^ bilayer model provides a reliable and reproducible tool for assessing host response in mucormycosis.

## Introduction

Invasive mycoses are responsible for significant morbidity and mortality in immuno-compromised patients. While *Aspergillus fumigatus* remains the most common cause of opportunistic mold infections, emerging fungal pathogens including those of the order Mucorales are observed with an increasing incidence (Roden et al., 2005). Invasive mucormycosis poses a serious clinical challenge, but its pathogenesis and immunopathology are still poorly understood (Roilides et al., 2014; Roilides and Simitsopoulou, 2017).

Humans are exposed to thousands of airborne mold spores every day and thus epithelial barriers of the respiratory tract, especially the 0.5–2 μm thin alveolar-capillary barrier with its over 100 m^2^ surface, commonly represent the primary site of interaction (Croft et al., 2016). In addition to their anatomical barrier function, airway epithelia demonstrably contribute to a fined-tuned immunological balance, which is required to control fungal invasiveness while preventing hypersensitivity, hyperinflammation, and excessive tissue damage (Park and Mehrad, 2009). Epithelial cells recognize fungal pathogens by TLR and C-type-lectin-receptor dependent pathways (Sun et al., 2012), which in turn triggers transcriptional changes including the upregulation of genes associated with inflammatory responses and damage repair (Cortez et al., 2006; Osherov, 2012). Infected airway epithelial cells release a broad array of antimicrobial peptides, enzymes, and cytokines, and thereby contribute to both fungal clearance and orchestration of their micro-environment through cytokine signaling (Park and Mehrad, 2009; Osherov, 2012).

Understanding the mechanisms of early fungal invasion and epithelial defense is a key to the development of novel treatment strategies, but also to the evaluation of diagnostic biomarkers (Hope, 2009; Kniemeyer et al., 2016). Increasingly complex *in vitro* models have been established in *Candida* and *Aspergillus* research to characterize host-pathogen interplay at epithelial barriers (Hope, 2009; Croft et al., 2016). Most commonly, an alveolar bilayer model has been employed in different studies of invasive aspergillosis to monitor invasion, host response, and diagnostic biomarkers (Hope et al., 2007; Gregson et al., 2012; Morton et al., 2014). This model consists of monolayers of human lung adenocarcinoma epithelial cells (A549) and human pulmonary artery endothelial cells (HPAEC) cultured on both sides of a Transwell^®^ membrane, allowing for separate analysis of cellular responses (e.g., transcriptional profiles) or soluble markers (e.g., cytokine concentrations) in each compartment. Refinement of the model was achieved by addition of different immune cell subsets (Morton et al., 2014), facilitating assessment of the interplay between epithelial cells and innate immunity. Particular focus was placed on the role of dendritic cells (Morton et al., 2014), as they serve as an important crosslink with the T-helper cell system and contribute to shaping adaptive immune response (Lass-Florl et al., 2013).

Recent reports revealed striking differences in innate immune response to *Aspergillus* and Mucorales (Warris et al., 2005; Chamilos et al., 2008; Wurster et al., 2017). For example, a strong pro-inflammatory response to Mucorales spores was observed, whereas resting *Aspergillus* conidia, covered by an immunologically inert hydrophobin layer, induce little inflammation (Wurster et al., 2017). The biological and clinical implications of these differences remain largely unexplored. To characterize the inflammatory response to Mucorales in the alveolar context, this study aimed to adapt and validate the alveolar bilayer Transwell^®^ model for Mucorales research, evaluate its technical reliability, and compare cytokine response, and epithelial barrier invasion upon infection with three common human pathogenic Mucorales species.

## Materials and Methods

### Pre-analytical Methods

#### PBMC Isolation and Generation of Monocyte-Derived Dendritic Cells (moDC)

Peripheral blood mononuclear cells (PBMC) were obtained by ficoll gradient centrifugation of leukocyte reduction systems kindly provided by the Institute for Transfusion Medicine and Immunohematology Wuerzburg. Subsequent monocyte isolation was conducted using MACS CD14 positive selection (Miltenyi Biotec). moDC maturation was induced by incubation with 20 ng/ml IL-4 and 100 ng/ml GM-CSF for 6 days at 37°C, 5% CO_2_.

#### Preparation of Molds

*Aspergillus fumigatus* ATCC 46645, *Rhizopus arrhizus* CBS 110.17, *Rhizomucor pusillus* CBS 245.58, and *Cunninghamella bertholletiae* CBS 187.84 were cultured on beer wort agar plates. Conidia were harvested by gently scraping cultures with a cotton swab, diluted in distilled water, and passed through a 40 μm cell strainer to remove residual mycelium. Spores were diluted at a concentration of 5 × 10^6^ cells per ml in endothelial cell basal medium 2 (EBM2) + 10% fetal calf serum (FCS) + endothelial cell growth medium 2 SingleQuots^TM^ without gentamicin/amphotericin B (GA-1000), hereinafter called “human pulmonary artery endothelial cell (HPAEC) medium.”

#### Preparation of Fungal Culture Supernatants

To obtain supernatants of fungal cultures, 1 × 10^7^ fungal spores were cultured in 2 ml EBM2 medium (Lonza) + 10% FCS in 6-well-plates for 30 h. Supernatants were sterile-filtered (0.2 μm) and cryopreserved at -20°C until further use. Thawed supernatants were diluted 1:1 with fresh medium before addition to the alveolar model.

#### Construction of Transwell^®^ Bilayer Model

An alveolar bilayer model was constructed as previously described (Hope et al., 2007; Hope, 2009). Briefly, human pulmonary artery endothelial cells (HPAEC, Lonza) and adenocarcinoma human alveolar basal epithelial cells (A549, Lonza) were cultured in 75 cm^2^ flasks in HPAEC medium + 0.1% GA-1000 at 37°C, 5% CO_2_. 1 × 10^5^ HPAECs were seeded on the lower side of a 6.5 mm diameter 3 μm pore Transwell^®^ membrane (Corning). After 2 h incubation, inserts were placed in HPAEC medium in a 24 well plate overnight. Subsequently, 5 × 10^4^ A549 cells suspended in 100 μl HPAEC medium were added to the upper compartment. Incubation of the assembly was performed in 24 well plates containing 600 μl HPAEC medium per well at 37°C, 5% CO_2_. Media changes were performed every other day. 50 μl moDC solution (2.5 × 10^5^ cells in HPAEC medium) or plain medium were added on day 5. Afterwards, inserts were infected by adding 50 μl fungal solution (2.5 × 10^5^ spores in HPAEC medium), followed by another 30 h incubation period at 37°C, 5% CO_2_. For selected experiments, 50 μg/ml gefitinib (Santa Cruz Biotechnology), 8 mg/ml D-(+)-glucose (Sigma-Aldrich), or 1 mg/ml beta-hydroxybutyrate (Sigma-Aldrich) were added to the endothelial compartment.

### Analytical Methods

#### Gene Expression Analyses

Endothelial cells and residual mycelium were removed from the lower side of the membrane using a cell scraper. After washing membranes in Hank’s Balanced Salt Solution (HBSS), inserts were transferred to wells containing 600 μl RNAprotect Cell Reagent (Qiagen) to preserve the total RNA of A549 cells ± moDCs. RNA isolation and cDNA synthesis were performed using the RNAeasy Mini Kit (Qiagen) and High Capacity cDNA Reverse Transcription Kit (Applied Biosystems) according to the manufacturer’s instructions.

#### Quantitative PCR

RT-qPCR was performed on a StepOne Plus PCR Cycler (Applied Biosystems) using the iTaq Universal SYBR Green Supermix (Bio-Rad) according to the manufacturer’s instructions. Initial denaturation (30 s at 95°C) was followed by 40 cycles of denaturation (3 s at 95°C) and annealing/elongation steps (30 s at 60°C). Melting curves were obtained at +0.5°C/15 s. The following primer sequences were used: *Cas3*: 5′-CTCTGGTTTTCGGTGGGTGT-3′ and 5′-TCCAGAGTCCATTGATTCGCT-3′; *Cas9*: 5′-CAGGCCCCATATGATCGAGG-3′ and 5′- TCGACAACTTTGCTGCTTGC-3′; *ICAM-1*: 5′-ACCCCGTTGCCTAAAAAGGA-3′ and 5′-AGGGTAAGGTTCTTGCCCAC-3′; *IL-6*: 5′-AAAGAGGCACTGGCAGAGAAAAC-3′ and 5′-AAAGCTGCGCAGAATGAGATG-3′; *IL-8*: 5′-AAGAAACCACCGGAAGGAAC-3′ and 5′-ACTCCTTGGCAAAACTGCAC-3′; *CCL2*: 5′-CCCCAGTCACCTGCTGTTAT-3′ and 5′-AGATCTCCTTGGCCACAATG-3′; *CCL5*: 5′-TCATTGCTACTGCCCTCTGC-3′ and 5′- TACTCCTTGATGTGGGCACG-3′; *Alas1* (house-keeping gene/reference gene): 5′-GGCAGCACAGATGAATCAGA-3′, and 5′-CCTCCATCGGTTTTCACACT-3′. Relative mRNA expression levels normalized to Alas1 were calculated using the ΔΔCt method.

#### Cytokine Analyses

Culture medium from both compartments was collected, centrifuged at 7,000 *g* for 5 min, and supernatants were frozen at -20°C until analysis. After 1:1 dilution with fresh HPAEC medium, cytokine concentrations in the culture supernatant were determined using a magnetic bead-based cytokine assay (13-plex HCYTOMAG, Merck Millipore) or TNF-α/IL-1β ELISA (ELISA Max Deluxe Kits, BioLegend) according to the manufacturer’s instructions.

#### 18S Ribosomal DNA PCR Assay

A Mucorales-specific real-time PCR assay (Muc18S) was used for semi-quantitative Mucorales DNA analysis as described before (Springer et al., 2016a,b). 0.5 ml of supernatant supplemented by 0.5 ml PBS was used for DNA extraction as described before (Springer et al., 2016b).

#### LDH Quantification

Culture supernatants from the upper compartment were diluted 1:5 in 0.9% NaCl. Lactate dehydrogenase (LDH) concentrations were determined by the central laboratory of the University Hospital of Wuerzburg using a diagnostic LDHI2 assay on a Cobas Integra analyzer.

#### Dextran Blue Assay

The alveolar model was assembled as described above and 1 μg dextran blue (5, 20, or 70 kDa) was added to the upper compartment. Inserts were placed in 600 μl HPAEC medium and incubated at 37°C, 5% CO_2_. After 30 min, 90 min, 5 h, 10 h, 20 h, and 30 h, inserts were transferred to a new well containing fresh HPAEC medium to prevent back-diffusion. Dextran blue concentrations in the previously used wells were quantified photometrically at 622 nm by comparison with standard dilutions.

#### Statistical Analysis

Unless otherwise stated, significance testing was performed using the paired or unpaired two-sided Student’s *t*-test. ^∗^*p* < 0.05, ^∗∗^*p* < 0.01, ^∗∗∗^*p* < 0.001. Though not considered significant, we provided an additional indication of *p* < 0.1 (■).

## Results

### Validation of an Alveolar Bilayer Model for Mucormycosis Research

**FIGURE 1:**
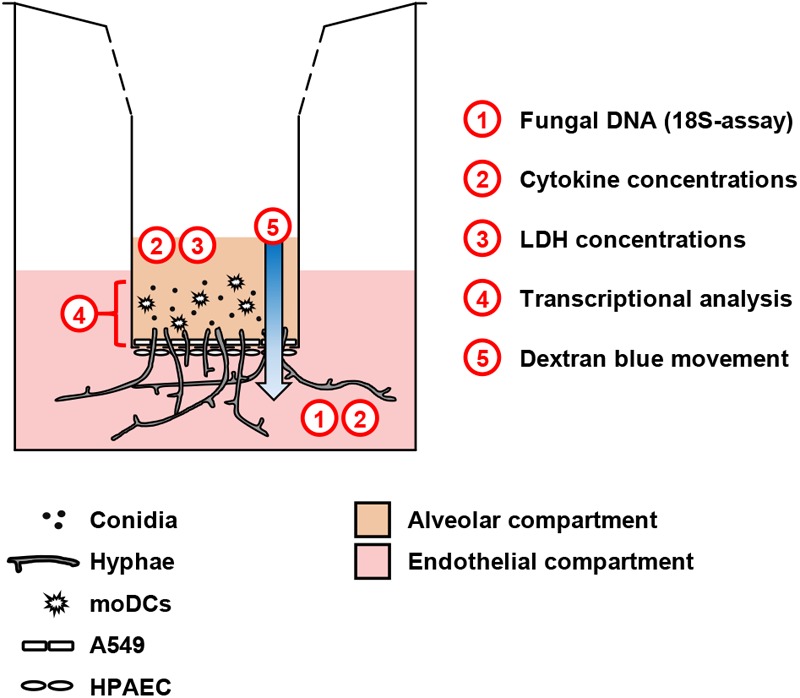
Schematic representation of the alveolar model. Human pulmonary endothelial cells (HPAEC, lower part) and type 2 pneumocytes (A549, upper part) were cultivated on a 3 μm Transwell^®^ membrane. Fungal cells and/or moDCs were added to the upper compartment. For selected experiments, additional agents (e.g., glucose) were added to the endothelial environment. The scheme summarizes readouts from each compartment.

**FIGURE 2:**
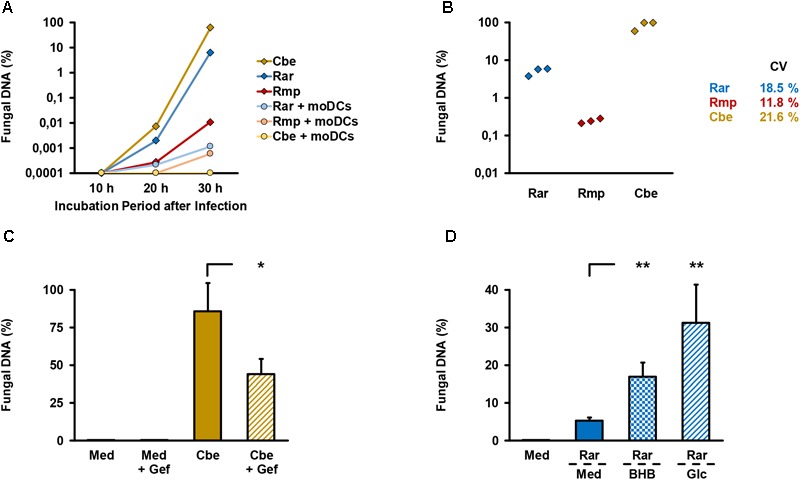
Validation of endothelial compartment invasion and its reproducibility. **(A)** The bilayer model was assembled as described in the Section “Materials and Methods”. 2.5 × 10^5^ spores of different Mucorales species ± 2.5 × 10^5^ moDCs were added to the upper compartment. Mucorales DNA in the lower compartment was quantified using an 18S-based assay. The diagram shows the percentage of detected DNA in the lower compartment depending on incubation time after infection of the upper compartment. The percent scale refers to a positive control (=100%) incubated for 30 h after addition of the same number of fungal spores directly to the lower compartment. Mean values based on technical duplicates from one representative run are shown. Rar, *R. arrhizus*; Rmp, *R. pusillus*; Cbe, *C. bertholletiae.* **(B)** To document the reproducibility of endothelial compartment invasion, the model was assembled and infected with spores of each pathogen in three independent experiments. Mucorales DNA was quantified after 30 h and compared with positive controls as described in **(A)**. Mean values of technical duplicates in each run are shown and inter-assay CVs are provided. Intra-assay CVs were consistently < 10%. **(C)** Endothelial compartment invasion by *C. bertholletiae* was assessed in the presence or absence of 50 μg/ml gefitinib (Gef) in the lower chamber. Uninfected inserts (medium control, Med) with and without gefitinib served as negative controls. Mucorales DNA was quantified after 30 h and compared with positive controls as described in **(A)**. Three independent experiments were performed. Mean values and standard deviations are shown. **(D)** Prior to infection with *R. arrhizus* (Rar), Transwell^®^ inserts assembled as described in the Section “Materials and Methods” were transferred to new wells containing either regular HPAEC medium (Med, glucose concentration: 1 mg/ml) or HPAEC medium supplemented with 8 mg/ml glucose (Glc) or 1 mg/ml beta-hydroxybutyrate (BHB). Mucorales DNA in the lower compartment was quantified 30 h post-infection and normalized to a positive control (=100%) as described above. Mean values from four independent experiments and standard deviations are shown. The two-sided Student’s *t*-test was used for significance testing in **(C,D)**. ^∗^*p* < 0.05, ^∗∗^*p* < 0.01.

An alveolar bilayer model was set up as described in the Section “Materials and Methods” and infected with three common human pathogenic Mucorales species to assess cytokine response and epithelial barrier integrity (Figure 1). A semi-quantitative 18S DNA assay was performed to evaluate epithelial barrier invasion by the studied Mucorales species, resulting in *trans*-epithelial growth. A constant increase in Mucorales DNA content in the lower compartment was detected over time (Figure 2A). Consistent with the macroscopic observation of more rapid and abundant mycelium formation, *R. arrhizus* and *C. bertholletiae* showed earlier and stronger increase in DNA content compared to *R. pusillus*. Addition of moDCs to the upper compartment effectively suppressed fungal invasion of the lower compartment, indicated by reduced Mucorales DNA content. To ensure sufficient fungal penetration of the alveolar barrier even in the presence of moDCs, an incubation period of 30 h was selected for subsequent experiments. Evaluating the inter-experiment reproducibility of endothelial compartment invasion in three independent experiments, coefficients of variation (CVs) were 11.8–21.6% (Figure 2B).

As an additional means of validation, we assessed *C. bertholletiae*, the most invasive isolate in our selection, to determine whether our bilayer model can recapitulate the finding of epithelial growth factor receptor (EGFR)-dependent A549 cell invasion recently described by others (Watkins et al., 2018). Addition of 50 μg/ml gefitinib, an FDA-approved EGFR inhibitor, to the endothelial chamber significantly reduced *C. bertholletiae* DNA content in the lower compartment after 30 h (Figure 2C, 86% versus 44% of positive control, *p* = 0.047). Moreover, enhanced endothelial invasion by *Rhizopus* in a hyperglycemic or ketoacidotic environment was previously shown in endothelial cell monolayers (Gebremariam et al., 2016). To validate the recapitulation of these findings in our model, we added 8 mg/ml glucose or 1 mg/ml beta-hydroxybutyrate to the endothelial compartment. Both treatments strongly and significantly (*p* < 0.01) increased the *R. arrhizus* DNA content in the endothelial compartment 30 h after inoculation of the alveolar chamber (Figure 2D).

### Mucorales Stimulate a Strong Pro-inflammatory Cytokine Response by moDCs, but Cause Reduced Chemokine Release From A549 Cells

**FIGURE 3:**
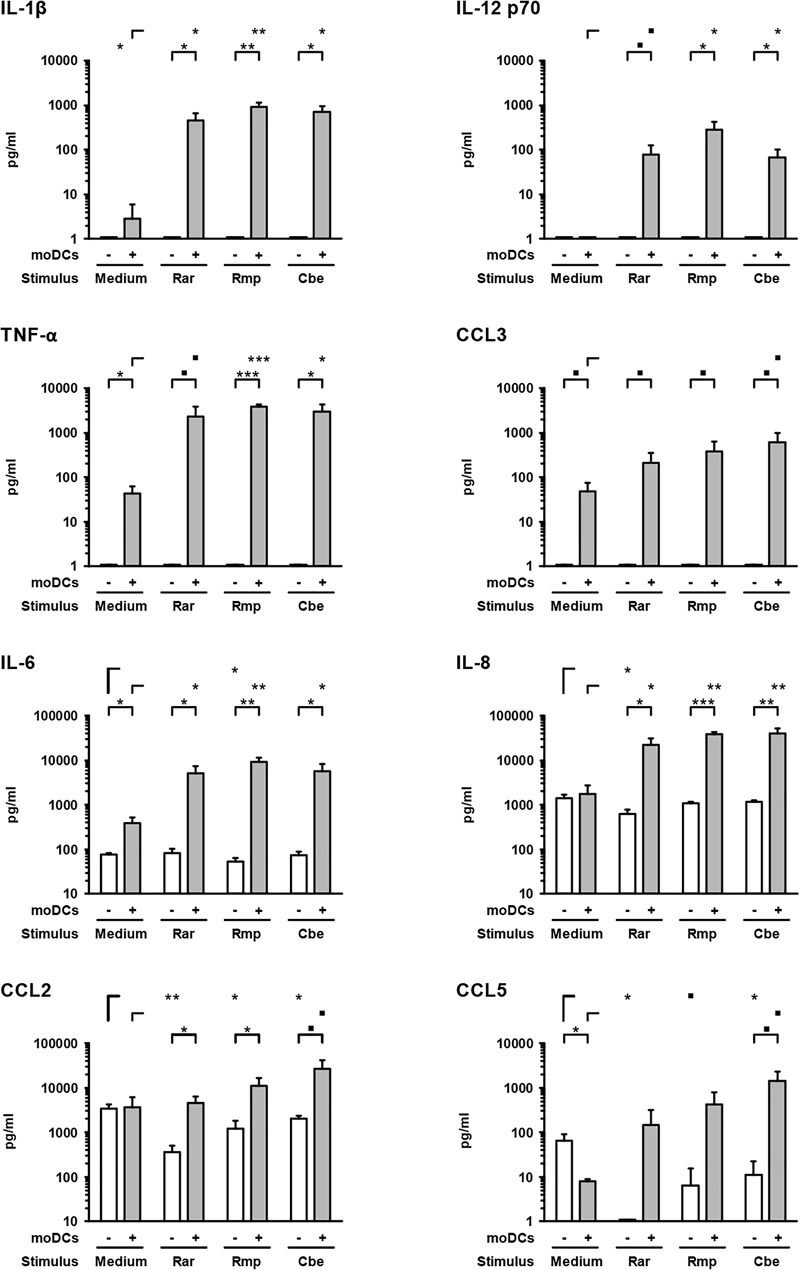
Mucorales stimulate strong pro-inflammatory cytokine response of moDCs. Concentrations of selected cytokines and chemokines were determined in the upper compartment using a 13-plex assay after 30 h of incubation with 2.5 × 10^5^ resting Mucorales spores or mock infection with medium (Med) in the presence or absence of moDCs. Mean values and standard deviations of four independent experimental runs using moDCs from different donors are shown. Two-sided Student’s *t*-test was used for significance testing. ■*p* < 0.1, ^∗^*p* < 0.05, ^∗∗^*p* < 0.01, ^∗∗∗^*p* < 0.001. Rar, *R. arrhizus*; Rmp, *R. pusillus*; Cbe, *C. bertholletiae*.

Next, we studied the cytokine response of epithelial cells and moDCs to Mucorales infection by applying a magnetic multiplex cytokine assay to culture supernatants from the alveolar (upper) compartment. Compared to uninfected samples, the secretion of pro-inflammatory cytokines IL-1β (161–335-fold), IL-6 (13–24-fold), IL-8 (12–23-fold), IL-12 p70 (78–289-fold), and TNF-α (54–89-fold) was strongly stimulated by the studied Mucorales species in the presence of moDCs (Figure 3).

**FIGURE 4:**
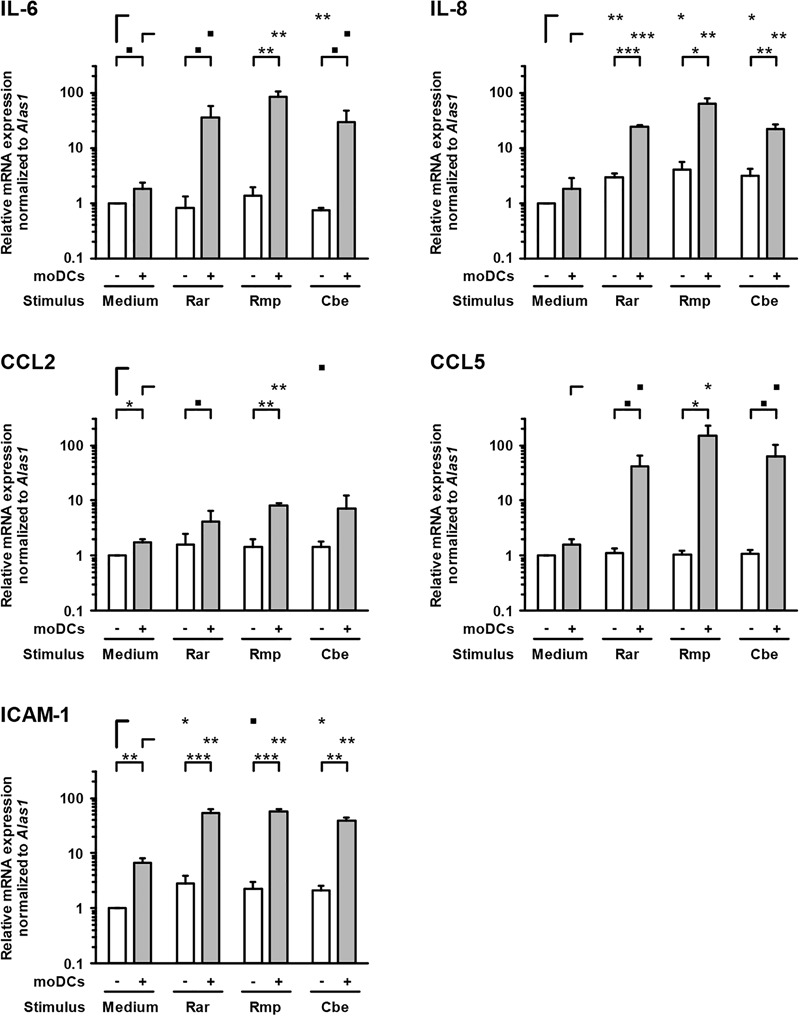
Transcription of epithelial cytokines, chemokines, and adhesion molecules. Relative mRNA expression of *IL-6, IL-8, CCL2, CCL5*, and *ICAM-1* in A549 ± moDCs was analyzed by RT-qPCR after 30 h of incubation with Mucorales spores or mock infection with cell-free medium (Med). Relative expression levels were normalized to *Alas1* using the ΔΔCt method. Mean values and standard deviations of four independent experimental runs using moDCs from different donors are shown. Two-sided Student’s *t*-test was used for significance testing. ■*p* < 0.1, ^∗^*p* < 0.05, ^∗∗^*p* < 0.01, ^∗∗∗^*p* < 0.001. Rar, *R. arrhizus*; Rmp, *R. pusillus*; Cbe, *C. bertholletiae*.

In samples lacking moDCs, IL-6 and IL-8 were also secreted by epithelial cells and remained largely unaffected by Mucorales exposure, except minor reductions of IL-6 and IL-8 secretion upon *R. arrhizus* and *R. pusillus* infection, respectively (Figure 3). Inversely, mRNA expression of IL-8 in A549 was significantly stimulated in infected samples (2.9–4.1, Figure 4). Mucorales infection also caused 2.1–2.9-fold induction of *Icam1* transcription in A549 and 5.9–8–4-fold upregulation in the presence of moDCs (Figure 4).

In inserts containing moDCs, CCL5 (RANTES) mRNA expression (26–92-fold) and secretion (19–180-fold) was stimulated by all tested pathogens compared to uninfected controls, though statistical significance was not attained for some of the treatments due to considerable inter-individual variation (Figure 4). In the absence of moDCs, the release of chemokines CCL2 and CCL5 from A549 cells was markedly reduced in Mucorales-infected samples (CCL2: 0.11–0.60-fold, CCL5: 0.02–0.17-fold, Figure 3). This observation was most pronounced for *R. arrhizus*, although transcriptional levels of *CCL2* and *CCL5* in A549 cells remained unaltered (Figure 4).

### Pro-inflammatory Cytokine Response Aggravates Alveolar Barrier Dysfunction Caused by Mucorales Infection

**FIGURE 5:**
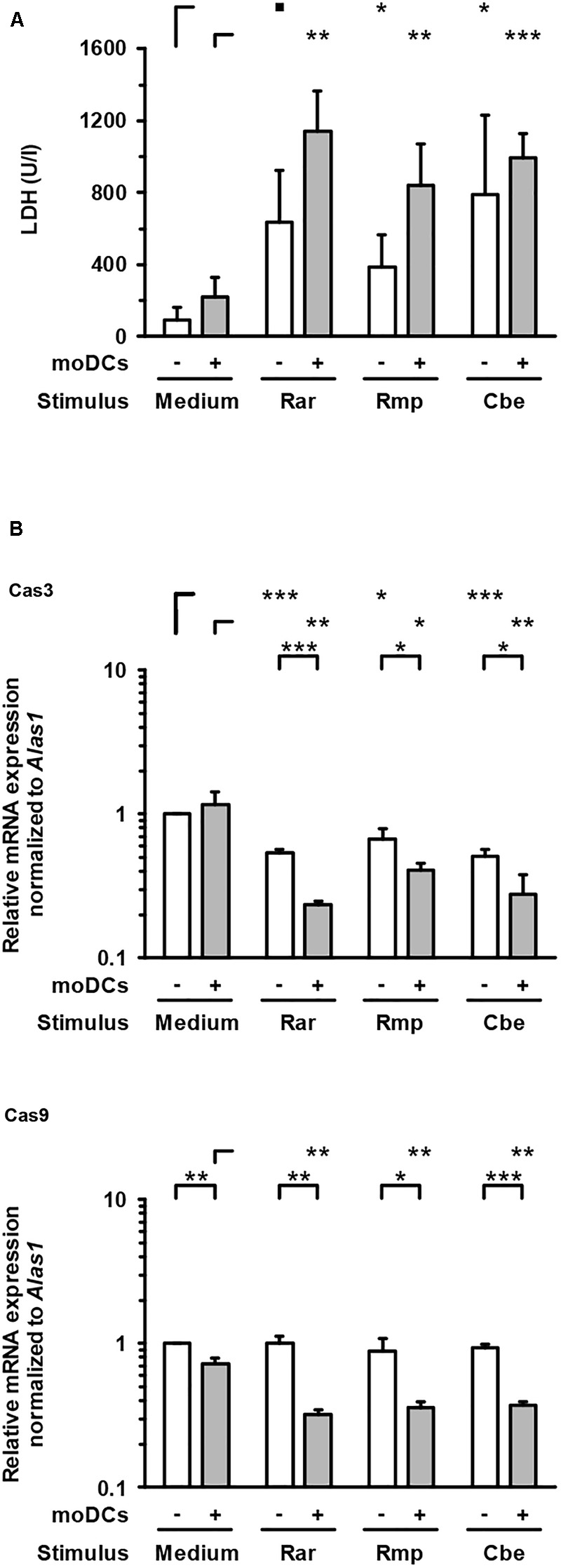
Lactate dehydrogenase concentrations and caspase gene expression in the epithelial compartment. **(A)** LDH concentrations in the upper compartment were quantified after 30 h of incubation with or without moDCs and/or Mucorales spores. **(B)** Relative mRNA expression of *Cas3* and *Cas9* in A549 ± moDCs was analyzed by RT-qPCR after 30 h of incubation with Mucorales spores or mock infection with cell-free medium (Med). Relative expression levels were normalized to *Alas1* using the ΔΔCt method. For both readouts, mean values and standard deviations of four independent experimental runs using moDCs from different donors are shown. Two-sided Student’s *t*-test was used for significance testing. ■*p* < 0.1, ^∗^*p* < 0.05, ^∗∗^*p* < 0.01, ^∗∗∗^*p* < 0.001. Rar, *R. arrhizus*; Rmp, *R. pusillus*; Cbe, *C. bertholletiae*.

To evaluate epithelial cell damage caused by Mucorales infection, LDH concentrations were quantified in culture supernatants of the upper compartment. Compared to uninfected control samples, upper chamber LDH levels in Mucorales-infected inserts were significantly increased (4.1–8.3-fold, Figure 5A). Addition of moDCs led to further enhancement of LDH release, though statistical significance was not reached for this observation. While *R. arrhizus* and *C. bertholletiae* caused greater LDH release in the absence of moDCs, the strongest increase in cytotoxicity was observed when moDCs were added to *R. pusillus* infected inserts (2.2-fold increase). By contrast, transcription of *Cas3* was suppressed in Mucorales exposed A549 cells compared to uninfected samples regardless of moDC addition, whereas *Cas9* mRNA expression was only significantly reduced in the presence of moDCs (Figure 5B).

**FIGURE 6:**
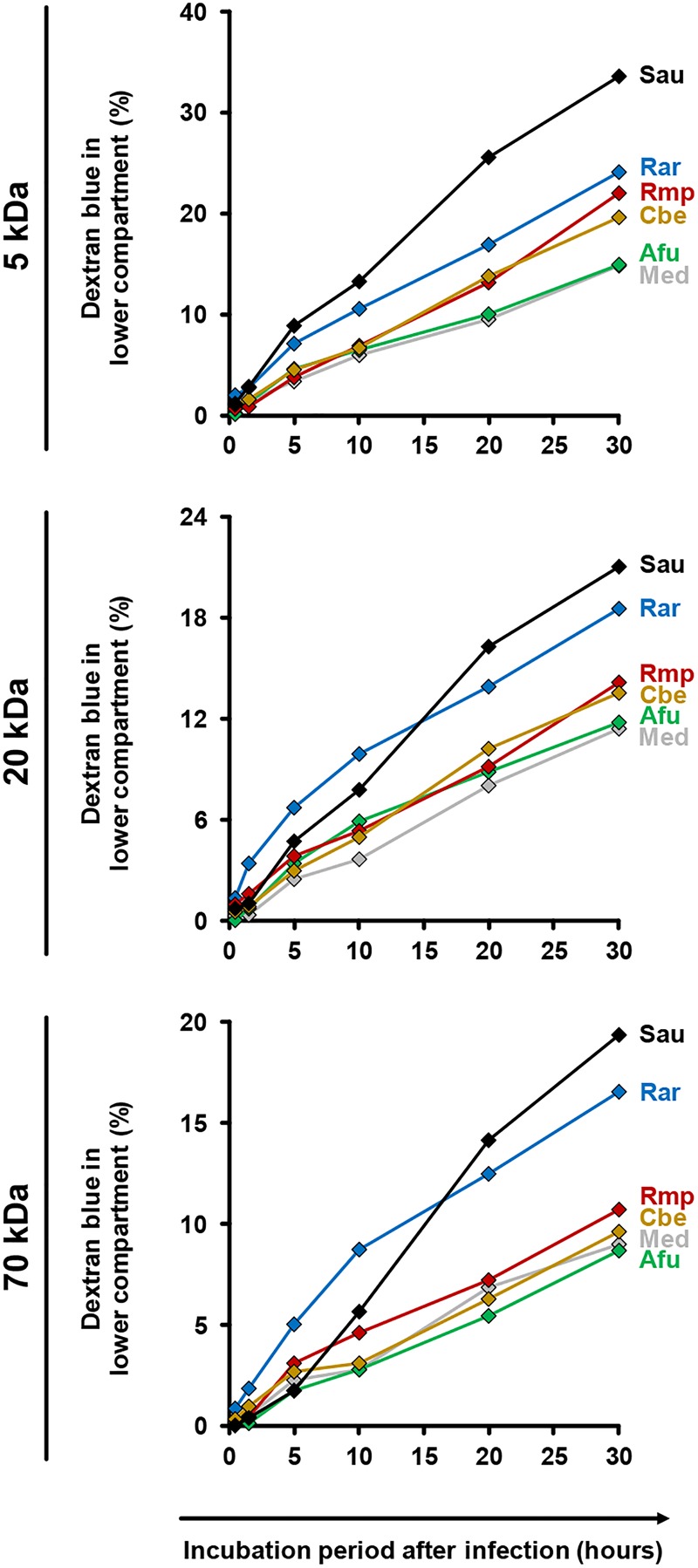
Impact of fungal infection on alveolar barrier permeability for macro-molecules. Culture inserts were prepared and infected as described in the Section “Materials and Methods.” 1 μg dextran blue (5, 20, or 70 kDa, diluted in HPAEC medium) was added to the upper compartment. Inserts were placed in 24 well plates containing 600 μl pre-warmed HPAEC medium per well. After 30 min, 90 min, 5 h, 10 h, 20 h, and 30 h inserts were transferred to a new well with fresh HPAEC medium to prevent back-diffusion. 100 μl medium from the previously used well were transferred to a 96 well microplate and absorbance was determined at 622 nm. Dextran blue concentrations were calculated by comparison of absorbance (OD) values with a standard curve of different dextran blue concentrations. The figure shows the percentage (cumulated over time) of dextran blue passing through the epithelial barrier (100% = 1 μg). The following stimuli were used: Medium (Med), *A. fumigatus* conidia (Afu), *Staphylococcus aureus* cells (Sau, clinical isolate), and Mucorales spores (Rar, *R. arrhizus*; Rmp, *R. pusillus*; Cbe, *C. bertholletiae*). Technical duplicates were analyzed and mean values are shown in the figure (CVs consistently < 15%).

For further assessment of epithelial barrier dysfunction, we performed a time-course analysis of membrane permeability using a dextran blue assay (Figure 6). Both uninfected inserts and those exposed to *A. fumigatus* showed minor *trans*-epithelial movement of dextran blue, reaching 9% (70 kDa) to 15% (5 kDa) after 30 h. Greater epithelial permeability was observed in the presence of Mucorales (10–17% for 70 kDa, 20–24% for 5 kDa). Among the studied Mucorales species, *R. arrhizus* caused the earliest and strongest increase in epithelial barrier permeability, most prominently for 70 kDa dextran blue.

**FIGURE 7:**
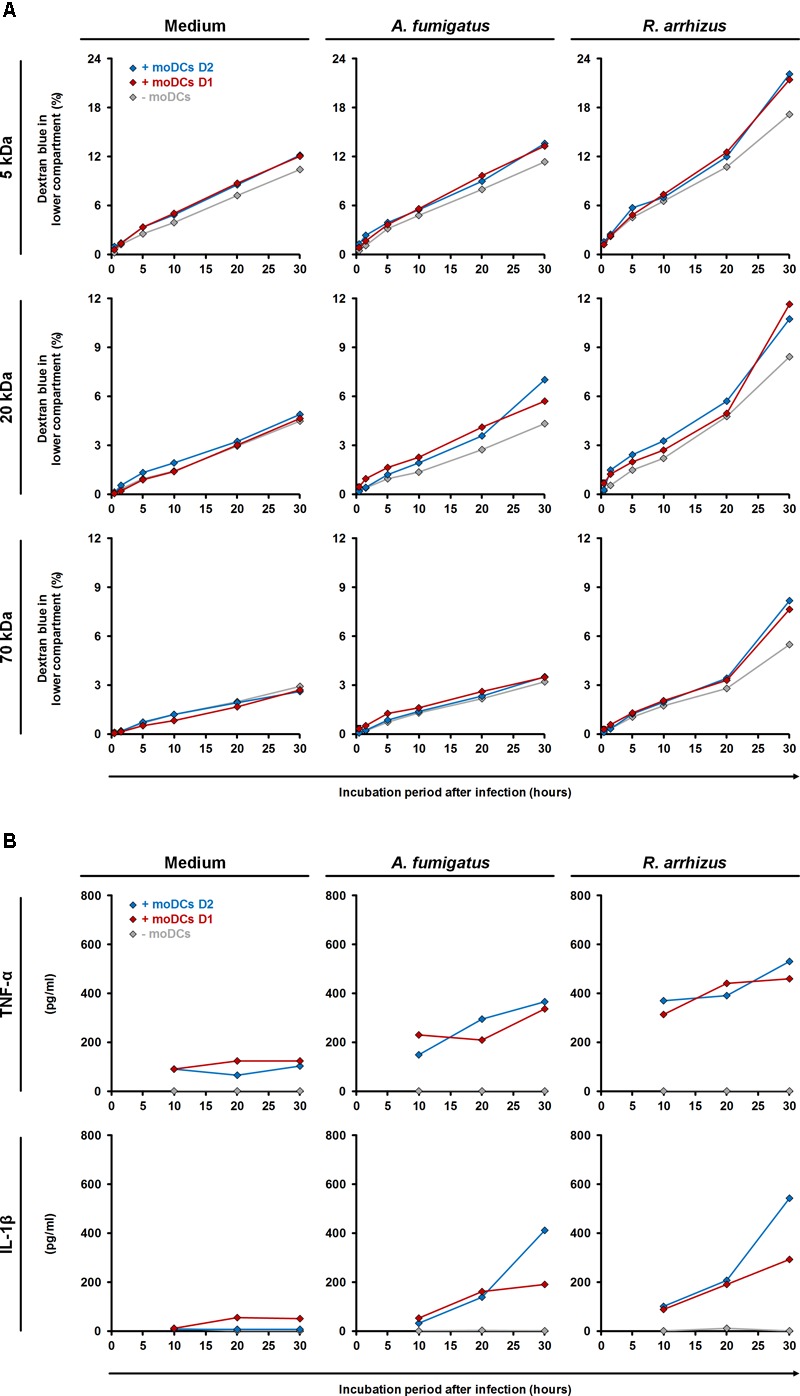
Influence of dendritic cells on alveolar barrier dysfunction caused by *A. fumigatus* and *R. arrhizus.* **(A)** Dextran blue assays were performed as described in the Section “Materials and Methods” and figure legend 6. 2.5 × 10^5^ moDCs from two healthy donors (red and blue diamonds) or plain medium (gray diamonds) were added to the dextran blue solution. Medium (left column), *A. fumigatus* conidia (central column), and *R. arrhizus* spores (right column) were used for infection. **(B)** In parallel, concentrations of TNF-α and IL-1β were determined in inserts without dextran blue 10, 20, and 30 h after infection. **(A,B)** Dextran blue assays and ELISA were performed in technical duplicates, and mean values are shown in the figure (CVs consistently < 20%).

To assess the impact of moDC-mediated inflammation, *trans*-epithelial movement of dextran blue was comparatively quantified in the presence and absence of moDCs (Figure 7A). *R. arrhizus* infection caused a 65% (5 kDa) to 88% (70 kDa) increase in dextran blue movement after 30 h compared to uninfected treatments and epithelial leakage was further aggravated by moDCs (+27 to +44%, Figure 7A). For *C. bertholletiae*, a more heterogeneous impact of moDCs was found, with an early increase in 5 and 70 kDa permeability, whereas *trans*-epithelial movement of 20 kDa dextran blue was only increased for one of the two donors tested (Supplementary Figure S1). While epithelial permeability was not affected by *A. fumigatus* alone (-3 to +9%), combined presence of the pathogen and moDCs resulted in minor elevations of *trans*-epithelial dextran blue movement (+10 to +46%, Figure 7A). TNF-α and IL-1β concentrations in both chambers were determined by ELISA (Figure 7B). Concordant with data presented in Figure 3, TNF-α and IL-1β release by moDCs was strongly increased in *R. arrhizus*-infected samples, whereas concentrations of these cytokines were 28–55% lower in *A. fumigatus*-infected samples.

To directly link inflammatory cytokines and epithelial damage, 1 ng/ml TNF-α and IL-1β were added to the upper chamber medium of Transwell^®^ inserts containing A549 and HPAEC, but neither fungal pathogens nor moDCs. Comparing *trans*-epithelial movement of dextran blue with control inserts not supplemented with cytokines, 23–37% higher concentrations of dextran blue were detected in the lower chamber after 30 h (Supplementary Figure S2). We also assessed whether secreted fungal metabolites contribute to epithelial damage. Addition of sterile-filtered supernatants from 30 h Mucorales cultures to the upper chamber of alveolar model inserts led to minimal LDH release (Supplementary Figure S3), suggesting that Mucorales-related epithelial damage is mostly due to rapid fungal invasion, aggravated by strong pro-inflammatory response of mononuclear cells.

### The Alveolar Bilayer Model Is a Highly Reproducible *in vitro* System for Mucormycosis Research

Apart from immunological research, the alveolar bilayer model was proposed as an *in vitro* tool for pharmacokinetic studies, antifungal drug screening, and investigation of new biomarkers (Hope et al., 2007). As these applications require highly reproducible experimental conditions, intra-assay, inter-assay, and inter-individual coefficients of variation of selected readout parameters were determined over a course of four independent experimental runs (Table 1). In the absence of Mucorales infection, median intra-assay CVs and inter-assay CVs for cytokine concentrations in the upper compartment ranged from 14.9 to 18.1% and 8.7 to 24.4%, respectively. Addition of fungal spores did not significantly influence intra-assay CVs, indicating excellent reproducibility of Mucorales infection in technical duplicates. While *C. bertholletiae* infection did not lead to elevated inter-assay CVs, there was a tendency toward slightly greater variability of A549 cytokine response to *R. arrhizus* and *R. pusillus* in independent experimental runs. Relative mRNA expression levels of epithelial cytokines showed higher inter-assay CVs than cytokine concentrations. For all three fungal pathogens, however, at least 8 out of 10 studied parameters reached an inter-assay CV of less than 40%. Predictably, for most studied parameters, inter-individual CVs using moDCs from four different donors exceeded technical variability of the assay.

**Table 1:** The table summarizes median intra-assay (two inserts per condition in each experimental run), inter-assay (four independent experiments without moDCs), and inter-individual (moDCs obtained from four different donors) coefficients of variation for selected readout markers depending on the fungal stimulus.

	Medium	*R. arrhizus*	*R. pusillus*	*C. bertholletiae*
**Median intra-assay CV (w/o moDCs)**				
IL-6 concentration in upper compartment	17.0%	16.3%	13.4%	18.1%
IL-8 concentration in upper compartment	18.1%	19.7%	4.5%	5.2%
CCL2 concentration in upper compartment	14.9%	26.3%	21.0%	15.8%
**Inter-assay CV (w/o moDCs)**				
IL-6 concentration in upper compartment	8.7%	26.4%	17.0%	17.7%
IL-8 concentration in upper compartment	19.5%	22.9%	6.2%	9.1%
CCL2 concentration in upper compartment	24.4%	39.6%	52.2%	15.6%
IL-6 mRNA expression comp. to Medium	n/a	58.8%	43.1%	9.2%
IL-8 mRNA expression comp. to Medium	n/a	17.6%	35.0%	33.3%
CCL2 mRNA expression comp. to Medium	n/a	57.7%	39.4%	22.8%
CCL5 mRNA expression comp. to Medium	n/a	18.8%	17.4%	14.4%
ICAM-1 mRNA expression comp. to Medium	n/a	33.9%	31.7%	22.7%
Cas3 mRNA expression comp. to Medium	n/a	6.2%	18.5%	13.0%
Cas9 mRNA expression comp. to Medium	n/a	13.1%	20.9%	5.7%
**Inter-individual CV (with moDCs from four different donors)**				
IL-6 concentration in upper compartment	38.8%	48.4%	23.3%	42.3%
IL-8 concentration in upper compartment	57.0%	38.2%	11.8%	27.8%
CCL2 concentration in upper compartment	67.3%	41.8%	53.7%	59.3%
IL-6 mRNA expression comp. to Medium	n/a	62.6%	34.6%	67.1%
IL-8 mRNA expression comp. to Medium	n/a	66.2%	61.4%	88.9%
CCL2 mRNA expression comp. to Medium	n/a	41.8%	25.0%	58.6%
CCL5 mRNA expression comp. to Medium	n/a	66.5%	54.1%	66.2%
ICAM-1 mRNA expression comp. to Medium	n/a	32.5%	20.3%	34.2%
Cas3 mRNA expression comp. to Medium	n/a	18.4%	28.0%	15.6%
Cas9 mRNA expression comp. to Medium	n/a	16.5%	9.3%	13.6%

*Relative mRNA expression refers to analyses of cell samples from the upper compartment (A549 ± moDCs). For CV determination of cytokine analyses, only cytokines with concentrations consistently > 10 pg/ml (including samples without moDCs or fungi) were considered*.

## Discussion

Opportunistic mold pathogens of the order Mucorales account for an increasing share of invasive infections in immunocompromised patients (Roden et al., 2005; Ibrahim and Kontoyiannis, 2013). The most common site of invasive mucormycosis in patients with hematological malignancies is the lung (Roden et al., 2005). In contrast to invasive aspergillosis, knowledge of pathogen-host-interplay in mucormycosis is still very limited (Roilides et al., 2014). Direct interaction studies revealed striking differences in the immunopathology of aspergillosis and mucormycosis (Warris et al., 2005; Chamilos et al., 2008; Wurster et al., 2017), especially in the inflammatory response to resting spores. While such analyses provide basic insights into fungal immunopathology, more sophisticated models are required to mimic the complexity of respiratory epithelia as the primary site of fungal invasion.

On the example of invasive aspergillosis, bilayer (Hope et al., 2007; Gregson et al., 2012; Morton et al., 2014) or monolayer (Morton et al., 2018) Transwell^®^ models of the human alveolus have been introduced to complement animal studies and basic cell culture experiments (Hope et al., 2007; Gregson et al., 2012; Morton et al., 2014). Building upon the extensive characterization and validation of these models in aspergillosis research, this study employed the alveolar bilayer model to comparatively assess the host response and epithelial integrity upon infection with different Mucorales species. The tested species were selected from the top seven causative agents of invasive mucormycosis (Lanternier et al., 2012) due to their biological heterogeneity in terms of spore diameter and mycelial morphology (Ribes et al., 2000). Fungal invasion of the lower compartment was documented using an 18S ribosomal DNA assay, since previously employed biomarkers (Hope et al., 2007; Hope, 2009; Gregson et al., 2012; Morton et al., 2014) such as galactomannan are not capable of detecting Mucorales (Lackner et al., 2014). High reproducibility of endothelial barrier invasion was documented (Figure 2B). Moreover, we have validated that our model is able to capture important hallmarks of Mucorales immunopathology such as EGFR-dependent alveolar barrier invasion (Watkins et al., 2018) and enhanced growth into the endothelial compartment in a hyperglycemic or ketoacidotic environment (Gebremariam et al., 2016), both previously demonstrated in monolayer studies and *in vivo* models.

In analogy with earlier, *A. fumigatus*-based studies (Hope et al., 2007; Gregson et al., 2012; Morton et al., 2014, 2018), fungal invasion of the lower chamber was significantly reduced upon addition of moDCs to the alveolar compartment. In planktonic culture, moDCs were found to phagocytose and kill a significant proportion of *A. fumigatus* spores (Wasylnka et al., 2005; Morton et al., 2011). Though the knowledge of dendritic cell-Mucorales interplay is very limited, we have demonstrated that both resting spores and germinated stages of *R. arrhizus* induce maturation marker upregulation and pro-inflammatory cytokine response in moDCs (Wurster et al., 2017). Others have previously shown that germinated Mucorales activate pro-inflammatory responses by moDCs in a Dectin-1-dependent manner (Chamilos et al., 2010). In addition, T-helper cell activation in PBMC-Mucorales co-culture was found suggesting that both spores and germ tubes are efficiently taken up and phagolysosomally processed by mononuclear phagocytes (Wurster et al., 2017). Therefore, it is likely that, similar to *A. fumigatus*, moDCs possess the ability to contribute to the clearance of Mucorales and thereby reduce fungal burden in the endothelial compartment.

Assessing cytokine release in A549-moDC co-culture in the bilayer model, a broad range of pro-inflammatory cytokines and chemokines was detected in Mucorales-infected inserts. Compared to a previous report of *A. fumigatus*-A549 interactions (Morton et al., 2018), markedly greater levels of IL-1β, IL-8, and TNF-α were found in response to the studied Mucorales. Similarly, induction of IL-8 and ICAM-1 transcription in A549-moDC-co-cultures was decidedly stronger than in an aspergillosis bilayer model (Morton et al., 2014), indicative of a more robust induction of pro-inflammatory cascades including the NF-κB pathway (Melotti et al., 2001). Direct comparison of IL-1β and TNF-α release in the present study confirmed earlier and significantly stronger induction in inserts infected with *R. arrhizus* compared to *A. fumigatus*, corroborating our previous observation of an early and robust pro-inflammatory cytokine response to both resting and germinated stages of Mucorales in planktonic culture (Wurster et al., 2017). Minor and mostly non-significant interspecies differences in cytokine and chemokine response patterns of epithelial cells and moDCs were observed between the studied Mucorales. While *R. pusillus* led to more prominent release of IL-6 and IL-12 p70, *C. bertholletiae* caused greater CCL2, CCL3, and CCL5 secretion from moDCs. This observation, which may be attributable to variable recognition of fungal cell wall constituents (Roilides et al., 2014), however, is outweighed by far more distinct differences between Mucorales and *Aspergillus* (Morton et al., 2018).

We employed moDCs for reasons of comparability with previous alveolar bilayer studies in invasive aspergillosis (Morton et al., 2014, 2018), their extensive characterization in mold immunopathology (Mezger et al., 2008; Morton et al., 2011; Lother et al., 2014), and availability in large quantities using standardized protocols. The pulmonary dendritic cell repertoire is, however, heterogeneous and its composition undergoes dynamic changes depending on the degree of inflammation (Kim and Lee, 2014). In the inflammatory state, moDCs are generated in the lung and are pivotal for pro-inflammatory cytokine response, phagocytosis of fungal spores, and T_H_1 cell priming, whereas conventional and plasmacytoid DCs are the dominant subsets in the steady state (Kim and Lee, 2014). Planktonic *in vitro* culture revealed specific roles of these subsets in the interplay with *A. fumigatus*, driven by distinct repertoires of pattern recognition receptors (Lother et al., 2014). Moreover, Morton et al. (2014) described distinct differences in cytokine gene induction in conventional DCs and moDCs exposed to *A. fumigatus* in the alveolar bilayer model. Therefore, future studies on Mucorales immunopathology may aim to comparatively elucidate the functional role of different DC subsets in the alveolar context.

Expectably, some of the studied cytokines showed baseline secretion from uninfected epithelial cells (e.g., IL-6 and IL-8), suggesting sufficient viability of A549 cells at the time of assessment. Though IL-8 mRNA expression in A549 was stimulated, there was no enhanced release in response to Mucorales. This observation is in line with an earlier study reporting minor stimulation of IL-6 and IL-8 release from airway epithelial cells infected with *A. fumigatus* (Zhang et al., 2005). Interestingly, in the absence of moDCs, our results document reduced concentrations of epithelial cytokines CCL2 and CCL5 in the alveolar compartment of Mucorales infected Transwell^®^ inserts, whereas transcriptional activity compared to a reference gene remained largely unaffected and proper ICAM-1 transcriptional response was observed. This may be suggestive of reduced numbers of vital, cytokine-producing epithelial cells upon infection. Accordingly, assessment of LDH, released from the cytosolic compartment of injured or dead cells (Chan et al., 2013), revealed significantly elevated concentrations upon Mucorales infection, whereas *A. fumigatus* caused minimal LDH release into the alveolar compartment. However, reduced expression of the key pro-apoptotic mediator *Cas3* in Mucorales-challenged A549 was observed, despite induction of apoptosis-driving cytokines (e.g., TNF-α). These findings may indicate active suppression of apoptosis in the presence of Mucorales, a mechanism previously described in *A. fumigatus* infected A549 and tracheal epithelial cells (Berkova et al., 2006).

Importantly, addition of moDCs led to strongly elevated LDH levels and epithelial barrier permeability, indicating aggravation of cellular stress due to inflammatory cytokines and mononuclear cell metabolites. In particular, TNF-α and IL-1β, strongly upregulated in Mucorales-exposed moDCs (Wurster et al., 2017), have been described to contribute to alveolar barrier dysfunction and A549 permeability (Tang et al., 2014; Cox et al., 2015). In the present study, time course experiments affirmed the link and temporal relationship between mononuclear cell cytokine secretion and a stronger moDC-associated increase in epithelial barrier disruption caused by infection with *R. arrhizus* compared to *A. fumigatus*.

Even in the absence of moDCs the studied Mucorales species caused stronger epithelial barrier disruption than *A. fumigatus*. Increased *trans*-epithelial movement of dextran blue was not only observed for *R. arrhizus*, known to rapidly produce abundant mycelium, but to a lesser extend also for *R. pusillus* despite its less extensive mycelial morphology (Ribes et al., 2000). While interstitial penetration upon adhesion of conidia to the basal lamina contributes to alveolar barrier invasion by both *A. fumigatus* (Wasylnka and Moore, 2000; Croft et al., 2016) and Mucorales (Bouchara et al., 1996), distinct means of invasion upon conidial attachment to epithelial cells have been described (Filler and Sheppard, 2006). Microscopic studies documented that *A. fumigatus* mostly grows horizontal to the epithelium (Escobar et al., 2016). *A. fumigatus* conidia internalized by A549 enter the phagolysosomal pathway, but some conidia germinate within epithelial cells, penetrate the cell membrane, and invade the extracellular space (Wasylnka and Moore, 2000; Wasylnka et al., 2005). Moreover, a recent study found that *A. fumigatus* hyphae traversed the bronchial epithelium through reorganization of the host actin exoskeleton, forming a tunnel that allows hyphae to enter the cells without disturbing their integrity (Fernandes et al., 2018). These mechanisms apparently foster immune evasion and fungal survival in the alveolar environment (Margalit and Kavanagh, 2015; Escobar et al., 2016). By contrast, destructive invasive growth and rapid induction of cell damage are hallmarks of mucormycosis (Ibrahim et al., 2005; Filler and Sheppard, 2006). Though the pathogens’ ability to produce an array of cytotoxic metabolites and lytic enzymes is well established (Binder et al., 2014; Ghuman and Voelz, 2017), our data did not reveal a prominent contribution of soluble mediators to epithelial cytolysis, suggesting that rapid mycelial growth of Mucorales, combined with an early and strong pro-inflammatory response by mononuclear cells, result in more pronounced epithelial cell damage and *trans*-epithelial movement of macromolecules.

An important limitation of this study is the continued use of A549 adenocarcinoma cells to mimic the alveolar side of the epithelial barrier. While this approach facilitates comparability with previous studies on *A. fumigatus* (Morton et al., 2014, 2018) and a recent Mucorales pathogenicity study in A549 monolayers (Watkins et al., 2018), specific properties of cancerous cells need to be considered, for example when assessing apoptosis markers (Gazdar et al., 2010; Croft et al., 2016). The use of cell lines also does not reflect the morphologic and genetic heterogeneity of respiratory epithelia. For this reason, the development of a perfused dynamic culture model with primary cells was recently reported and applied to study mold immunopathology (Chandorkar et al., 2017). On the other hand, the A549 cell line demonstrably contributes to highly reproducible performance of the model with intra- and inter-assay CVs for cytokine concentrations and RT-qPCR based readouts consistently below 40%, allowing for the detection of inter-individual differences in innate immune response to the fungus, e.g., in studies assessing the influence of mutations in pathways associated with immune recognition of fungal pathogens.

In summary, this study presents a cost-effective and reliable *in vitro* model of mucormycosis, facilitating the screening of an array of pathogens, experimental conditions, or immune cell samples in parallel. Our findings reveal an early and strong pro-inflammatory response by dendritic cells, which aggravates epithelial barrier dysfunction. Due to its high reproducibility and ability to capture important hallmarks of Mucorales immunopathology, the alveolar bilayer model may become an appealing tool for the *in vitro* screening of antifungal leads and biomarkers related to host immunity.

## Data Availability Statement

Preliminary data of this study have been presented at the European Congress of Clinical Microbiology and Infectious Diseases 2017, Vienna, Austria.

## Ethics Statement

The use of anonymized leukocyte reduction chambers from platelet apheresis systems did not require approval by an Ethics Committee or written informed consent.

## Author Contributions

SB and LP performed the experiments and data analysis and contributed to the manuscript. ML, MD, and JS performed the experiments. AW-G analyzed the data. CM contributed to study design, data analysis, and manuscript preparation. HE, JL, and AU provided discussions, and contributed to study supervision and manuscript preparation. SW designed and supervised the study, performed experiments and data analysis, and wrote the manuscript.

## Conflict of Interest Statement

The authors declare that the research was conducted in the absence of any commercial or financial relationships that could be construed as a potential conflict of interest.
