# Supplementary material for: Comparative Analysis of Inflammatory Cytokine Release and Alveolar Epithelial Barrier Invasion in a Transwell® Bilayer Model of Mucormycosis
Source: Front Microbiol. 2019 Jan 8;9:3204. doi: 10.3389/fmicb.2018.03204 (PMC6332705; doi:10.3389/fmicb.2018.03204)
Supplement: Supplementary file 1 [file Data_Sheet_1.docx]

**Comparative analysis of inflammatory cytokine release and alveolar epithelial barrier invasion in a Transwell^®^ bilayer model of mucormycosis**

**Supplementary Figures**

**Supplementary Figure 1**

Dextran blue assays were performed as described in the Methods section and figure legend 6 and inserts were infected with 2.5 x 10^5^ *C. bertholletiae* spores. 2.5 x 10^5^ moDCs from two healthy donors (red and blue diamonds) or plain medium (grey diamonds) were added to the dextran blue solution. Technical duplicates were performed, and mean values are shown. CVs were consistently below 25 %.

**Supplementary Figure 2**

Trans-epithelial dextran blue movement was assessed as described in the methods section. Dextran blue was dissolved either in plain HPAEC medium (grey diamonds) or medium supplemented with 1 ng/ml TNF-α and IL-1β (black diamonds). The analysis was performed in duplicates, and mean values are shown. CVs were consistently below 10 %.

**Supplementary Figure 3**

Supernatants of fungal cultures (Rar = *R. arrhizus*, Rmp = *R. pusillus*, Cbe = *C. bertholletiae*, Afu = *A. fumigatus*) were generated and diluted as described in the methods section. Control supernatants (Ctrl) did not contain fungal pathogens. 100 µl of diluted supernatants were added to 100 µl A549 culture supernatants in the upper chamber. LDH concentrations in the upper compartment were quantified after 30 hours of incubation. Mean values and standard deviations based on two independent experimental runs are shown. *S. aureus* infected inserts were used as positive control (Sau).
